# Dependence of Electrical Conductivity on Phase Morphology for Graphene Selectively Located at the Interface of Polypropylene/Polyethylene Composites

**DOI:** 10.3390/nano12030509

**Published:** 2022-02-01

**Authors:** Ce Tu, Kenji Nagata, Shouke Yan

**Affiliations:** 1 State Key Laboratory of Chemical Resource Engineering, Beijing University of Chemical Technology, Beijing 100029, China; tuce@bgi-graphene.com (C.T.); skyan@mail.buct.edu.cn (S.Y.); 2 Department of Materials Science and Engineering, Graduate School of Engineering, Nagoya Institute of Technology, Nagoya 466-8555, Japan

**Keywords:** electrical conductivity, graphene, polypropylene (PP), polyethylene (PE), thermally reduced graphene oxide, PP/PE polymer blend, percolation threshold, processing sequence, melt blending

## Abstract

Conductive composites of polypropylene (PP) and polyethylene (PE) filled with thermally reduced graphene oxide (TRG) were prepared using two different processing sequences. One was a one-step processing method in which the TRG was simultaneously melt blended with PE and PP, called TRG/PP/PE. The second was a two-step processing method in which the TRG and the PP were mixed first, and then the (TRG/PP) masterbatch was blended with PE, called (TRG/PP)/PE. The phase morphology and localization of the TRG in TRG/PP/PE and (TRG/PP)/PE composites with different PP/PE compositions were observed by transmission electron microscopy (TEM) and scanning electron microscopy (SEM). The TRG was found to be selectively dispersed in the PE phase of the TRG/PP/PE composites, resulting in a low percolation threshold near 2.0 wt%. In the (TRG/PP)/PE composites, the TRG was selectively located at the PP/PE blend interface, resulting in a percolation threshold that was lower than 1.0 wt%. With the addition of 2.0 wt% TRG, the (TRG/PP)/PE composites exhibited a wide range of electrical conductivities at PP/PE weight ratios of 10 w/90 w to 80 w/20 w. Moreover, electrical and rheological measurements of the composites revealed that the co-continuous phase structure is the most efficient candidate for the fabrication of conductive composites.

## 1. Introduction

Graphene is an atomically thin, 2-dimensional network of sp^2^-hybridized carbon atoms and is expected to be a promising replacement for other carbon-based fillers (e.g., carbon black, graphite and carbon nanotubes) in polymer composites, owing to its superior electrical, thermal, and mechanical properties [1,2]. Particularly, its high aspect ratio makes graphene very effective in endowing various properties to polymer matrices, especially in the field of conductive polymer composites. The graphene-filled polymer composite, a functional composite material, has therefore attracted much attention due to its potential applications in several fields, such as antistatic materials [3], auto-regulating resistors, electrodes [4], electromagnetic interference shielding [5,6], and polymer pigmentation.

Electrical conductivity can be significantly improved by the addition of only a small concentration of graphene to polymers [7,8]. Very low percolation thresholds have been achieved for various types of insulating matrices; for example, 0.5 wt% reduced graphene oxide (RGO) in poly(vinyl alcohol) [9], and 0.2 wt% in PS-functionalized graphene sheet (FGS) composites [10].

To achieve the lowest possible percolation and obtain optimal properties of the composites, the proper selection of graphene-based filler, polymer matrix, and preparation method (e.g., melt blending, solution mixing and in situ polymerization) is a key factor. One effective method is to control the state of the dispersion and distribution of graphene in multiphase polymer composites by making the graphene selectively locate in one phase or at the interface of an immiscible blend. Qi et al. [11] prepared a PS/PLA/graphene composite that showed a percolation threshold of 0.15 wt% caused by the selective localization of graphene in the PS phase, compared with a percolation threshold of 0.7 wt% for PS/graphene binary composites. Such a significant decrease in the percolation threshold was attributed to the double percolation phenomenon first proposed by Sumita et al. [12]. This double percolation concept was also applied in our previous work [13], in which we investigated PP/PE/thermally reduced graphene oxide (TRG) composites and observed that the TRG became selectively located in the PE phase, resulting in a lower percolation threshold (3 wt%) compared with that of TRG-filled PE composites (10 wt%).

In addition to the localization of fillers in polymer blends, the phase morphology of the composite also plays an important role in the electrical properties of conductive additive-filled polymer composites [14]. Xu et al. [15] explored the phase morphology and electrical conductivity of PCL/PLA/acid-oxidized MWNT (A-MWNT) composites with PCL compositions of 5 to 90 wt%. They found that the A-MWNTs were selectively located in the PCL phase at various PCL compositions. The maximum electrical conductivity was observed at the PCL composition of 40 wt%, which was explained as caused by the formation of a co-continuous phase morphology and an optimal A-MWNT content. Mao et al. [16] investigated the effect of phase morphology on the electrical properties of PS/ MMA/octadecylamine-functionalized graphene (GE-ODA) composites and observed that their electrical conductivity was optimal when the PS and PMMA phases formed a co-continuous structure (50 w/50 w), in which the GE-ODA were selectively dispersed within the PS phase.

Although considerable effort has been devoted to investigating the effect of morphology on the electrical conductivity of graphene filled binary polymer composites [17], there have been few systematic investigations on graphene-filled immiscible polymer blends in which the graphene is located at the blend interface, which is a more efficient strategy for decreasing the graphene percolation threshold. For PLA/EVA/RGO composites, Shen et al. [18] reported a percolation threshold of 0.18 wt%, owing to the localization of graphene at the interface, 2.4 times lower than that obtained when the graphene was localized in the EVA phase. TRG has been found to become located at the interface of PP/PE blends when TRG/PP is mixed first and then mixed with PE, which leads to a transformation of the blends from insulating to conducting with a smaller percolation. Therefore, in the present work, PP/PE immiscible blends were selected as matrices to be filled with TRG. The main aim of this paper was to systematically investigate the dependence of the electrical properties of the TRG filled PP/PE composites on their phase morphology and TRG localization. Transmission electron microscopy (TEM) was used to visualize the phase morphologies of the composites, and their electrical conductivity was measured to establish a relationship between their electrical properties and phase morphology.

## 2. Materials and Methods

### 2.1. Materials

The high-density polyethylene (HDPE; HI-Zex, 2100J) used in this study was supplied by Mitsui Chemicals Inc. (Tokyo, Japan). The polypropylene (PP; NOVATEC-PP, MA3) was a commercial product of Japan Polypropylene Co. (Tokyo, Japan). Pristine expanded graphite flakes (EC300), with an average size of 50 μm, were supplied by Ito Graphite Co., Ltd. (Mie, Japan). Concentrated sulfuric acid (95−98%), fuming nitric acid (85%), hydrochloric acid (37%), and potassium chlorate (98%) were provided by Nacalai Tesque. Inc. (Kyoto, Japan). 

Graphene oxide was prepared from natural graphite by oxidation with KClO_3_ in concentrated H_2_SO_4_ and HNO_3_ according to Staudenmaier’s method [19,20]. Thermally reduced graphene oxide (TRG) was prepared by thermal reduction and exfoliation of the graphite oxide at ~1050 °C for ~30 s in a muffle furnace FT-101 (FULL-TECH Furnace Co., Ltd., Osaka, Japan). The detailed characterization of the obtained TRG was confirmed in our previous study [21].

### 2.2. Composite Preparation

The PP, PE, and TRG were dried at 50 °C under vacuum for 24 h before melt blending. TRG-filled PP/PE composites were prepared by melt blending in a HAAKE MiniLab conical twin-screw extruder at 190 °C and a rotational speed of 50 rpm under N_2_ atmosphere. The samples for electrical testing were compression-molded into 0.5-mm-thick films at 190 °C and 2.2 MPa.

Different localizations of the TRG were designed using two different blending sequences:

- TRG/PP/PE: The PP, PE, and TRG were melt-blended simultaneously for 7 min;
- (TRG/PP)/PE: The PP was first mixed with TRG for 2 min, and the resulting TRG/PP masterbatch was blended with PE for 5 min.

To investigate the influence of phase morphology on the electrical properties of the composites, both types of composites were tuned by adjusting the component ratios of the two polymers. For example, the PP composition varied from 0 to 100 wt%.

### 2.3. Characterization

#### 2.3.1. Transmission Electron Microscopy

TEM measurements were carried out on a JEM-z2500 (JEOL Ltd., Tokyo, Japan) at an accelerating voltage of 200 kV to observe the localization of the TRG and the phase morphologies of the composites. Ultrathin sections of the TRG filled PP/PE composites of about 100 nm in thickness were cut with a diamond knife at −140 °C on a microtome (LEICA EM UC7). Each ultrathin section was put on an elastic carbon-support film grid for TEM observation.

#### 2.3.2. Scanning Electron Microscopy

SEM measurements were performed on a JSM-6010LA (SEM, JEOL Ltd., Tokyo, Japan) at accelerating voltage of 10 kV. Samples were prepared by fracturing in liquid nitrogen and subsequent sputter-coating with a thin layer of platinum using an Auto Fine Coater (JEC-3000FC, JEOL Ltd., Tokyo, Japan).

#### 2.3.3. Electrical Properties

Electrical resistivity measurements (two-point probe array) were performed at room temperature and an input voltage of 10 V. An Advantest R8340A Ultra High Resistance Meter (Advantest Co., Tokyo, Japan) was used to test compression-molded samples prepared to a dimension of ϕ40 mm × 0.5 mm (diameter × thickness). The surface of the samples was painted with conductive silver paste to ensure good contact with the measurement electrodes. 

#### 2.3.4. Rheological Measurements

The rheological properties of the composites were measured with a VAR-50 rheometer (Jasco Co., Tokyo, Japan) using a parallel plate (diameter of 25 mm). A frequency sweep from 0.01 to 90 rad s^−1^ was carried out at 190 °C in under N_2_ atmosphere. The measurements were performed in dynamic mode with the gap set at 0.85 mm. Shear stress was maintained at 100 Pa throughout the experiments.

## 3. Results and Discussion

### 3.1. Composites Morphology

**Figure 1:**
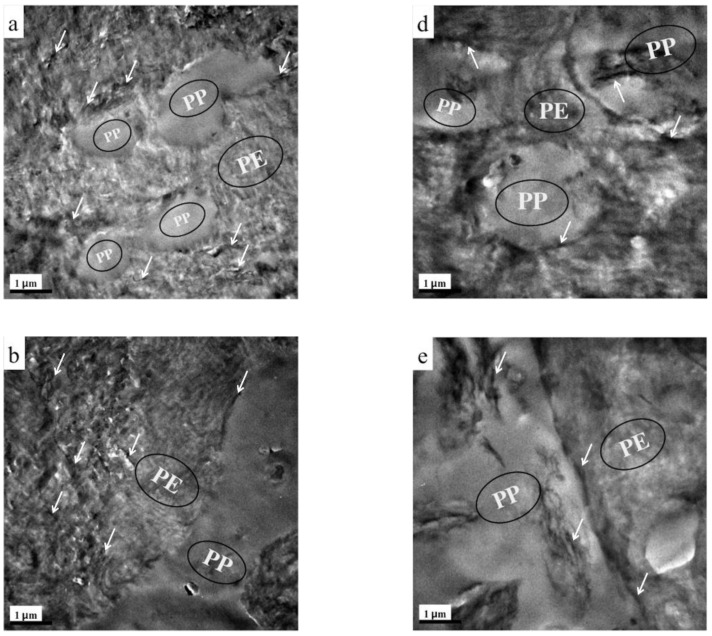

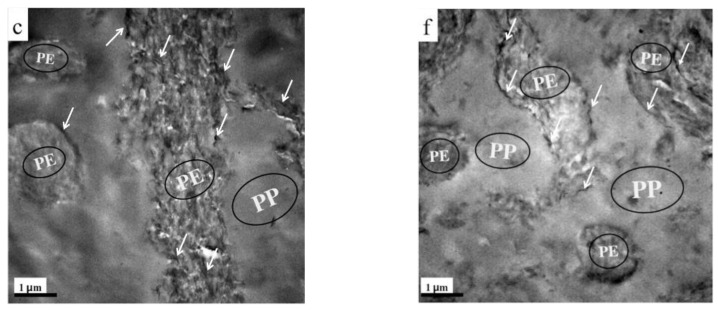
TEM images of (**a**) 30 w/70 w, (**b**) 50 w/50 w, and (**c**) 70 w/30 w TRG/PP/PE composites, and (**d**) 30 w/70 w, (**e**) 50 w/50 w, and (**f**) 70 w/30 w (TRG/PP)/PE composites. The content of TRG was 2.0 wt% in all samples. The white arrows indicate TRG.

The phase morphology and localization of TRG in the composites were characterized by TEM. Figure 1 presents the TEM images of TRG/PP/PE and (TRG/PP)/PE composites (2.0 wt% TRG) with different PP/PE compositions. A typical two-phase structure can be observed in the images, in which the dark and light areas correspond to the PE and PP phases, respectively [22]. From Figure 1a–c, it is clear that most of the TRG was selectively dispersed in the PE phase of the TRG/PP/PE composites, whether the PP was the discrete phase or the continuous phase. The selective localization of TRG in the PE phase was mainly caused by the lower interfacial tension between the PE and TRG compared with that between PP and TRG [13]. The morphology of the TRG/PP/PE composites could be tuned by adjusting the composition ratio of PE and PP. A typical sea-island structure was observed for the 30 w/70 w PP/PE blend (Figure 1a), i.e., spherical PP domains were dispersed in the continuous PE matrix. When the PP content was increased to 50 wt% (Figure 1b), a well-developed co-continuous structure formed. In the 70 w/30 w PP/PE blend (Figure 1c), the PE became a discrete phase, forming sphere or layer-like structures in the PP phase. The layer-like structure maintained the continuity of PE phase in the composite, while PP became the continuous phase.

Figure 1d–f show TEM images of (TRG/PP)/PE composites of various compositions. The change in the morphology of the (TRG/PP)/PE composites with PP/PE composition was similar to that of the TRG/PP/PE composites, from a sea–island structure at 30 wt% PP (Figure 1d) to co-continuous phases at 50 wt% PP (Figure 1e), and then back to the sea–island structure at 70 wt% PP (Figure 1f). In all of the (TRG/PP)/PE composites, the TRG sheets were randomly dispersed in both the PE and PP phases. However, notably, some of TRG sheets were clearly selectively located at the interface of the PP/PE blend, whatever the PP/PE morphology. This selective interfacial localization meant that the TRG sheets were well connected with one another, which should facilitate direct electron transport. This condition is regarded as an ideal scenario for obtaining the lowest possible electrical percolation threshold [8,18].

**Figure 2:**
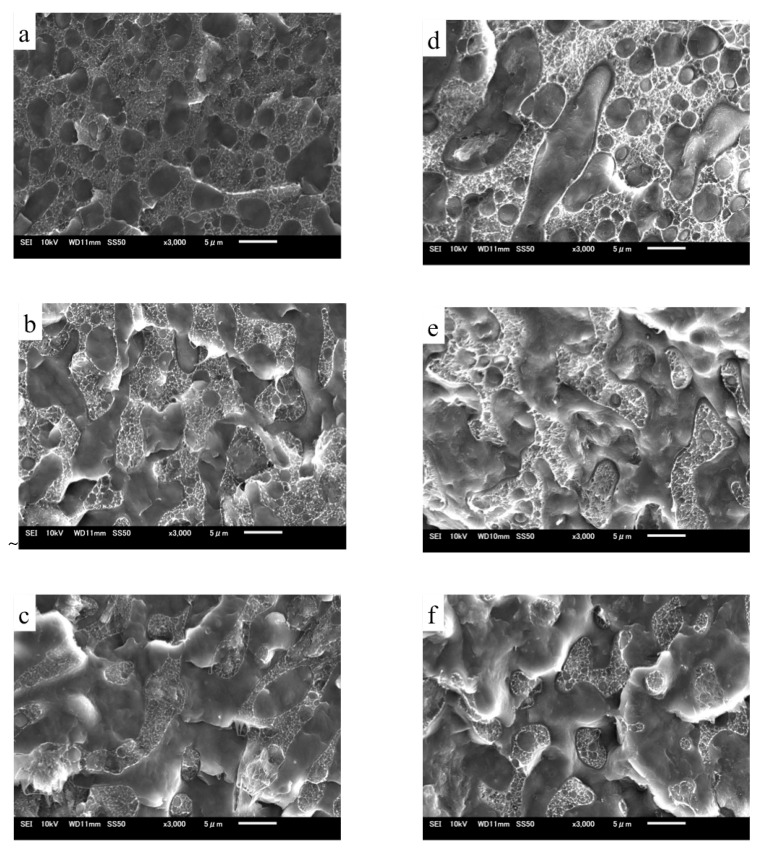
SEM micrographs of (**a**) 30 w/70 w, (**b**) 50 w/50 w, and (**c**) 70 w/30 w TRG/PP/PE composites, and (**d**) 30 w/70 w, (**e**) 50 w/50 w, and (**f**) 70 w/30 w (TRG/PP)/PE composites. The content of TRG was 2.0 wt% in all samples.

SEM observations were also performed to determine the phase morphologies of the TRG/PP/PE and (TRG/PP)/PE composites. Figure 2 shows SEM micrographs of fractured surfaces of TRG/PP/PE and (TRG/PP)/PE composites of different compositions. The rough part in each image corresponds to the PE phase, owing to its higher crystallinity, while the smooth part corresponds to the PP phase. The TRG/PP/PE and (TRG/PP)/PE composites showed similar changes in morphology with composition. At a PP content of 30 wt% (Figure 2a,d), the rough PE matrix was a continuous phase, and contained many dispersed droplet-like minor PP phase domains. For the 50 w/50 w PP/PE blends (Figure 2b,e), the PE and PP phases formed a co-continuous morphology. At a PP content of 70 wt % (Figure 2c,f), discrete minor PE components were observed in the PP matrix. However, compared with the (TRG/PP)/PE 70 w/30 w composite, which exhibited many minor PE spheres dispersed in a continuous PP phase (Figure 2f), the 70 wt% PP TRG/PP/PE composite contained a larger amount of the co-continuous-like PE structure coexisting with the island-like PE structure (Figure 2c). Similar results were observed for other PP/PE compositions; the PP domains were larger in the (TRG/PP)/PE composites than in the TRG/PP/PE composites at same composition. The above TEM observations revealed the differing localization of the TRG in the PP/PE blends: Most of the TRG was selectively dispersed in the PE phase of the TRG/PP/PE composites but was randomly dispersed in both the PE and PP phases of the (TRG/PP)/PE composites. This caused the TRG/PP/PE composites to have a relatively higher PE melt viscosity owing to the higher TRG concentration of the PE phase, which enlarged the phase continuity of the PE domains in these composites [23,24]. The roughness of the fractured surface of the PP domains observed in Figure 2d–f was greater than that in Figure 2a–c. This indicates that more TRG was distributed in the PP phase in the (TRG/PP)/PE composites. Moreover, larger cavities along the interface were observed in the (TRG/PP)/PE composites, which may have been caused by some of the TRG located at the PP/PE blend interface (shown in Figure 1d–f). 

### 3.2. Effects of Phase Morphology and Localization of TRG on Electrical and Rheological Properties

**Figure 3:**
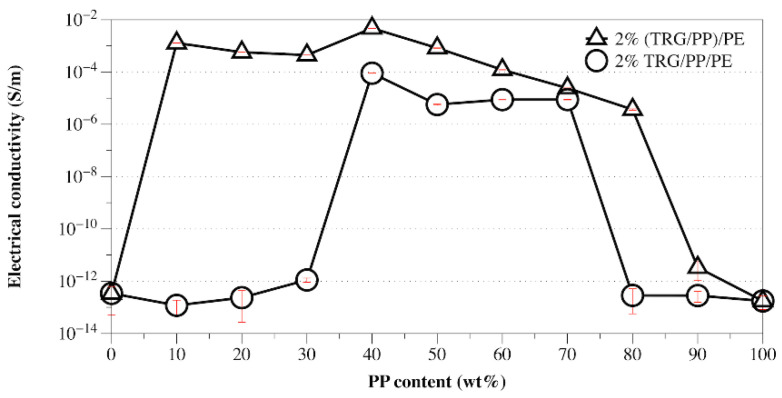
Electrical conductivity versus PP content for TRG/PP/PE and (TRG/PP)/PE composites. The content of TRG in the polymer blends was 2.0 wt%.

**Figure 4:**
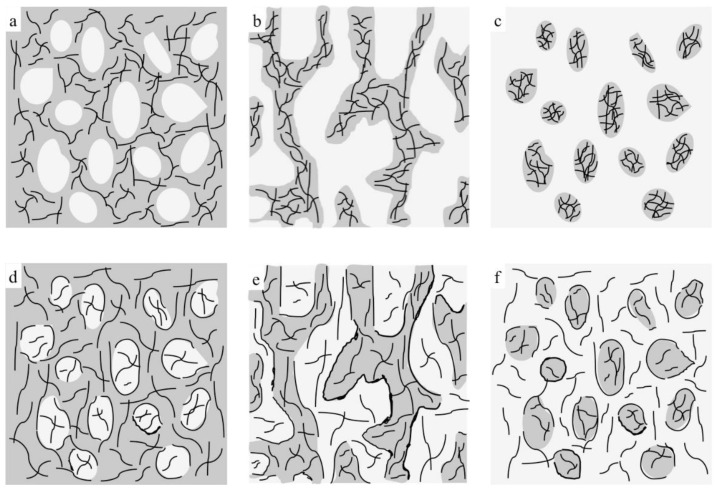
Schematic representations of morphological changes for (**a**–**c**) TRG/PP/PE and (**d**–**f**) (TRG/PP)/PE composites, in which gray regions represent PE phase, white regions represent PP phase, and black lines represent TRG.

From the above TEM and SEM observations, it is clear that the phase morphology of the present composites could be easily tuned by adjusting the composition ratio of PE and PP, and different processing methods led to different TRG localization behaviors. It is well known that the phase morphology of the composite and the localization of the filler are key factors that determine the electrical performance of polymer composites [25,26]. Figure 3 shows the change in electrical conductivity with PP content measured for the TRG/PP/PE and (TRG/PP)/PE composites. The content of TRG in the polymer blends was 2.0 wt%. The schematics displayed in Figure 4 aim to explain the observed changes in electrical conductivity based on the morphological TEM and SEM observations (Figure 1 and Figure 2).

When TRG was added to single PE matrix (0 wt% PP), the resulting TRG/PE composite displayed insulating properties, which indicated that the TRG content (2.0 wt%) was lower than its electrical percolation threshold in the PE matrix. In the TRG/PP/PE composites with a PP content between 10 wt% and 30 wt%, the PP formed a large number of spheres dispersed in a continuous PE phase, as shown in Figure 4a. Because the TRG highly selectively dispersed in the PE phase, an increase in PP composition, i.e., decrease in PE composition, led to an increase in the nominal TRG content in the PE phase relative to its overall content in the PP/PE composite. As a result, a slight increasing trend in conductivity was observed with increasing PP composition. A further increase in the PP content to 40 wt% resulted in a remarkable improvement in the electrical conductivity of the composite, about seven times greater than that of the 30 wt% PP composites, indicating that the TRG percolated networks were well developed for efficient electron transport. This significant improvement in electrical conductivity could be attributed to the fact that the TRG/PP/PE composites formed a co-continuous phase structure at a PP/PE weight ratio of 40/60 (Figure 4b), which caused a superior volume-exclusion effect of the PP [27]. Furthermore, greatly reducing the PE content from 100 to 60 wt% caused a significant increase in the nominal concentration of TRG in the PE phase, which allowed the TRG to form a better-percolated conductive network throughout the PE phase at the same overall loading of TRG. As the PP content was further increased to 50 and 70 wt%, so did the nominal TRG concentration in the PE phase. Although a declining trend appeared in the continuity of the PE phases, the TRG-filled PE phase maintained its connectivity throughout the PP phase (Figure 2c). As a result, the conductivity of these composites showed a slight decrease but remained high, owing to the formation of the conductive network. When the PP content was increased beyond 80 wt%, the conductivity of the composites dropped remarkably because the TRG-filled PE phase domains became insulated by the continuous PP phase and so could not form a percolated network throughout the composites (Figure 4c). These results indicate that the transition region of the PE phase from continuous to discrete phase in the TRG/PP/PE composite was between 20 wt% and 30 wt% PE. When TRG was added to neat PP, the resulting composite was insulating, indicating that the TRG concentration (2.0 wt%) was below its electrical percolation threshold in the PP matrix.

In contrast, the (TRG/PP)/PE composites displayed a wide range of electrical conductivities at PP/PE weight ratios of 10 w/90 w to 80 w/20 w, but all exhibited markedly better electrical conductivity than that of the TRG/PP/PE composites of the same PP/PE composition. This superior performance is attributed to the selective localization of TRG at the interface of the PP/PE blends. The interface-localized TRG tended to become more or less ordered along the interface of the PP/PE blends during melt mixing, as observable in Figure 1d–f. In contrast to the case of disorderly TRG dispersion, this interfacial localization of the TRG caused the interface to become TRG-rich, which enhanced filler-filler interactions in the interfacial region and thus promoted the formation of the percolated TRG network even at a lower electrical percolation threshold [28,29]. In the (TRG/PP)/PE composites with a PP content of 10 wt% or 30 wt%, some of the TRG was ordered along the surface of the PP spheres, well-located in the interface, as shown in the schematic illustration in Figure 4d. This resulted in a tremendous improvement in electrical conductivity compared with that of the corresponding TRG/PP/PE composites. Meanwhile, the 40 w/60 w PP/PE composite showed the maximum electrical conductivity, which could be attributed to the co-continuous structure having the best interfacial continuity of the binary blends [14]. This led to more TRG becoming located at the interface, making the conductive network form more easily, as illustrated in Figure 4e. When the content of PP was increased to 70 wt% or 80 wt%, as shown in Figure 4f, discrete PE spherical domains were dispersed in the continuous PP matrix, and the TRG enwrapped the discrete PE domains well. These composites still showed high electrical conductivity because the interface localized TRG was connected to the TRG dispersed in the PE and PP phases, helping to build electrical pathways throughout the composites. Upon a further increase in PP content to 90 wt%, the resulting decrease in the interfacial area with the size of the discrete PE phases made the conductive network more difficult to form, and the composite became insulating.

**Figure 5:**
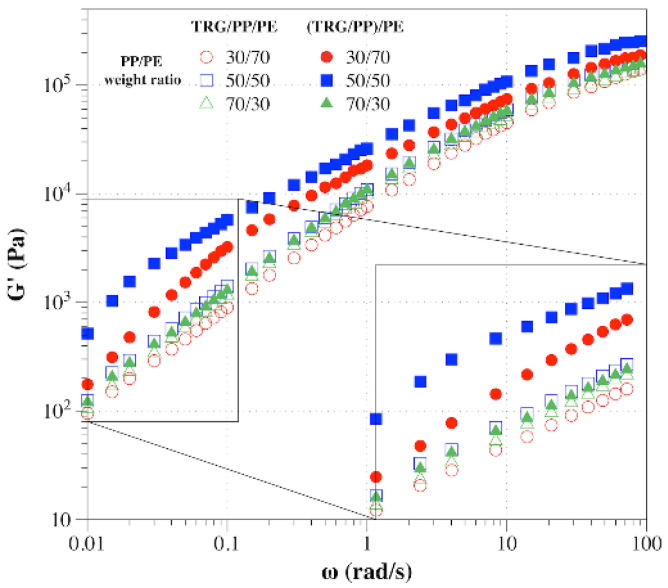
Plots of storage modulus (*G’*) versus frequency for TRG/PP/PE and (TRG/PP)/PE composites with various PP/PE compositions. The content of TRG in the polymer blends was 2.0 wt%.

To further investigate how the phase morphology and localization of TRG affected the formation of the TRG network, the rheological behaviors of the composites were characterized, which is an effective method of detecting the formation of percolated filler networks [30,31]. The storage modulus (*G’*) measured during the frequency sweep of TRG/PP/PE and (TRG/PP)/PE composites of different PP to PE weight ratios is shown in Figure 5. For TRG/PP/PE, the 50/50 composite showed a slightly higher *G’* than that of the 70 w/30 w composite, whereas the 30 w/70 w composite exhibited the lowest *G’*. Because the conductivity of the 50 w/50 w composite was close to that of the 70 w/30 w composite, the low-frequency *G’* of the former was slightly higher than that of latter, mainly owing to the better continuity of its TRG filled PE phase, as shown in Figure 2b,c. In contrast, the rheological properties of the (TRG/PP)/PE composites worsened in the order: 50 w/50 w, 30 w/70 w, 70 w/30 w. This tendency of the rheological properties was in good accordance with the electrical properties of the composites. More importantly, the *G’* of the (TRG/PP)/PE composites was higher than that of the TRG/PP/PE composites of the same PP/PE composition, with a particularly strong difference observed for the 50 w/50 w and 30 w/70 w blends. This indicated that the TRG more easily built a percolating TRG network in the (TRG/PP)/PE composites, in agreement with the morphology and electrical performance results.

### 3.3. Migration of TRG in the (TRG/PP)/PE Composites

**Figure 6:**
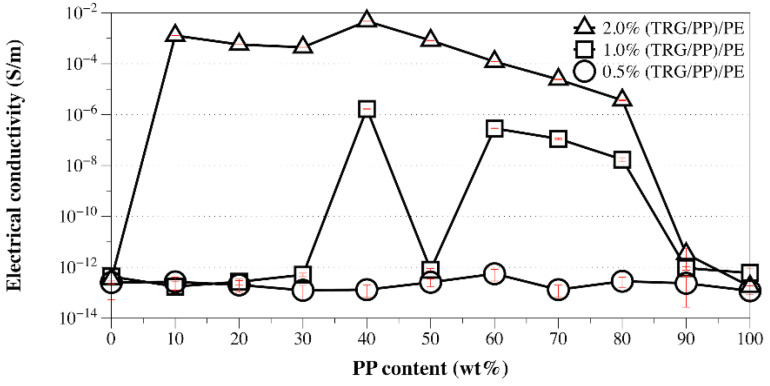
Electrical conductivity versus PP content of 0.5 wt%, 1.0 wt%, and 2.0 wt% TRG filled (TRG/PP)/PE composites.

It is known that fillers localized at the blend interfaces not only benefit the electrical conductivity but also lower the electrical percolation threshold of a composite [32]. Because the (TRG/PP)/PE composites showed a higher electrical conductivity than that of TRG/PP/PE composites at 2.0 wt% TRG, a lower electrical percolation threshold of the (TRG/PP)/PE composites was greatly expected. Figure 6 displays the dependence of the electrical conductivity on the PP content of the (TRG/PP)/PE composites as a function of their TRG loading. The electrical conductivity of the (TRG/PP)/PE composites with different PP/PE weight ratios generally increased with their TRG loading. At a TRG loading of 0.5 wt%, all the composites showed very low conductivity, which means that this loading of TRG (0.5 wt%) was below its electrical percolation threshold. The 1.0 wt% TRG filled composites conducted electricity when their PP content was between 40 wt% and 80 wt%, except for the 50 w/50 w PP/PE composition. Considering that the TRG was first mixed with the less favorable PP phase, a migration of TRG from the PP phase to the PE phase occurred during the second mixing step—the mixing of the (TRG/PP) masterbatch with PE—owing to the thermodynamic driving force. Thus, the localization of TRG at the blend interface strongly depended on the melt-mixing time of the (TRG/PP) masterbatch and PE [33]. The insulating property of the 50 w/50 w composite indicated that the localization of TRG at the interface of the 50 w/50 w PP/PE blend was less developed. Therefore, a more comprehensive understanding of the migration of TRG in the (TRG/PP)/PE 50 w/50 w composite needed to be investigated. 

**Figure 7:**
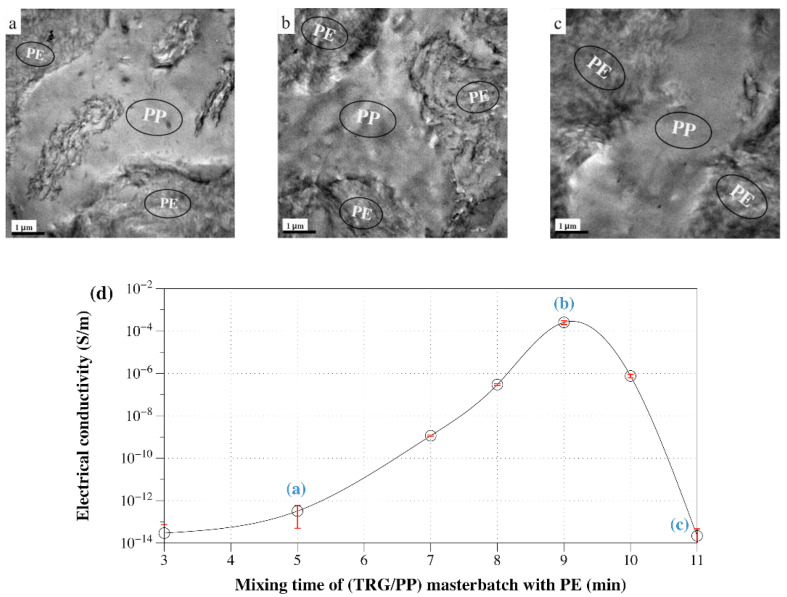
TEM micrographs of 1.0 wt% TRG filled (TRG/PP)/PE composites with different mixing time of the TRG/PP masterbatch with PE: (**a**) 5 min, (**b**) 9 min, and (**c**) 11 min. (**d**) Electrical conductivity versus mixing time of the (TRG/PP) masterbatch with PE for 1.0 wt% TRG filled (TRG/PP)/PE 50 w/50 w composite.

The dependence of the electrical conductivity of 1 wt% filled (TRG/PP)/PE 50 w/50 w composite with the mixing time of the TRG/PP masterbatch with the PE is shown in Figure 7. Figure 7a displays the phase morphology of the 50 w/50 w composites of Figure 6. A portion of the TRG was still essentially in the PP phase at short mixing time (≤5 min), and an interface localized TRG network did not develop, resulting in low conductivity. At longer mixing times, the composite became increasingly conductive. This observation is consistent with the migration of the TRG from the PP phase to the PE phase through the interface. The electrical conductivity passed through a maximum when most of the conducting TRG was concentrated in the interfacial region of the PE/PP blend (Figure 7b), thus a more well-developed conductive network formed at the same loading of TRG. Beyond 10 min of mixing, the composite again became highly insulating owing to the complete migration of the TRG into the PE phase (Figure 7c), in which its nominal content (2.0 wt %) was below the TRG percolation threshold.

Thus, the percolation threshold of the (TRG/PP)/PE composites was likely in the range of 0.5 wt% and 1.0 wt%, approximately half that of the TRG/PP/PE composites (near 2.0 wt%). Moreover, it should be pointed out that the 1.0 wt% (TRG/PP)/PE composites with co-continuous structure (40 w/60 w to 60 w/40 w PP/PE) exhibited higher electrical conductivity than that of the other composites. This indicates that the co-continuous phase is the most efficient candidate for the development of TRG filled conductive composites when the TRG is located at the interface.

## 4. Conclusions

The dependence of the electrical properties of TRG filled PP/PE composites with different blending sequences and various PP/PE compositions on their phase morphology and TRG localization have been systematically investigated. In TRG/PP/PE composites, the TRG selectively dispersed in the PE phase, while in the (TRG/PP)/PE composites the TRG localized at the PP/PE blend interface. For both types of composites, the formation of a co-continuous phase structure resulted in the effective development of electrical conductivity. The electrical percolation threshold of the (TRG/PP)/PE composites was in the range of 0.5 wt% to 1.0 wt%, much lower than that of the TRG/PP/PE composites (2.0 wt%). At a TRG content beyond the percolation threshold, the 2.0 wt % TRG filled (TRG/PP)/PE composites exhibited a wide range of electrical conductivities at PP/PE weight ratios of 10 w/90 w to 80 w/20 w. This result was attributed to the contact of the interface localized TRG with the TRG dispersed in the other phases, which aided the formation of a conductive network throughout the composite. When the TRG was only dispersed in the PE phase rather than concentrated at the interface, electrical conductivity was never observed when the PE phase was the isolated phase. The rheological properties of the composites were also measured to detect the percolated formation of TRG and were in accordance with the observed electrical properties and the phase morphologies. Additionally, the localization of TRG at the PE/PP blend interface was optimized by controlling the mixing time of the (TRG/PP) masterbatch with the PE. Our results convincingly emphasize that the localization of TRG at the blend interface makes the conductive network easier to develop and provides a way to fabricate conductive composites with various polymer blend phase morphologies.

## Data Availability

The data presented in this study are available on request from the authors.
